# The Isolation, Differentiation, and Survival In Vivo of Multipotent Cells from the Postnatal Rat *filum terminale*

**DOI:** 10.1371/journal.pone.0065974

**Published:** 2013-06-06

**Authors:** Ruchira M. Jha, Ryan Chrenek, Laura M. Magnotti, David L. Cardozo

**Affiliations:** 1 Department of Neurobiology, Harvard Medical School, Boston, Massachusetts, United States of America; 2 Department of Genetics, Harvard Medical School, Boston, Massachusetts, United States of America; University of Montréal and Hôpital Maisonneuve-Rosemont, Canada

## Abstract

Neural stem cells (NSCs) are undifferentiated cells in the central nervous system (CNS) that are capable of self-renewal and can be induced to differentiate into neurons and glia. Current sources of mammalian NSCs are confined to regions of the CNS that are critical to normal function and surgically difficult to access, which limits their therapeutic potential in human disease. We have found that the *filum terminale* (FT), a previously unexplored, expendable, and easily accessible tissue at the caudal end of the spinal cord, is a source of multipotent cells in postnatal rats and humans. In this study, we used a rat model to isolate and characterize the potential of these cells. Neurospheres derived from the rat FT are amenable to *in vitro* expansion in the presence of a combination of growth factors. These proliferating, FT-derived cells formed neurospheres that could be induced to differentiate into neural progenitor cells, neurons, astrocytes, and oligodendrocytes by exposure to serum and/or adhesive substrates. Through directed differentiation using sonic hedgehog and retinoic acid in combination with various neurotrophic factors, FT-derived neurospheres generated motor neurons that were capable of forming neuromuscular junctions *in vitro*. In addition, FT-derived progenitors that were injected into chick embryos survived and could differentiate into both neurons and glia *in vivo*.

## Introduction

Both neural stem cells (NSCs), which are capable of unlimited self-renewal, and neural progenitor cells (NPCs), which have a more restricted potential, hold great promise for cell replacement strategies to treat neurological diseases [1]. The hope is for these cells to replace those lost due to a disease process or trauma [2], [3]. In order for this strategy to be successful, NPCs have to produce the appropriate cell types in appropriate numbers and must integrate into the host nervous system to reproduce the correct circuitry. In addition, the transplanted cells cannot lead to tumor formation or initiate an immunological reaction.

The presence of multipotent NSCs has previously been demonstrated in multiple regions of the adult mammalian CNS in species ranging from rats to humans. These regions include the olfactory bulb [4], subependymal lining of the ventricles [5]–[9], hippocampus [10], [11], cerebellum [12], [13], spinal cord [14], [15], and retina [16], [17]. Recent studies have used isolated NSCs to generate specific neuronal types *in vitro*, such as retinal [18]–[21], dopaminergic [22]–[25], and cholinergic neurons [26]–[28]. In addition, NSCs transplanted into mammalian brains have retained their ability to generate various cell lineages and have survived *in vivo* [29]–[32].

Despite rapid advances in NSC biology, current sources of mammalian NPCs are not ideal for transplantation therapy in human disease because they are obtained from regions that are difficult to access and are critical to normal brain function. Surgical disruption of these areas leads to profound neurological deficits, which renders their use impractical for harvesting autologous NPCs.

**Figure 1:**
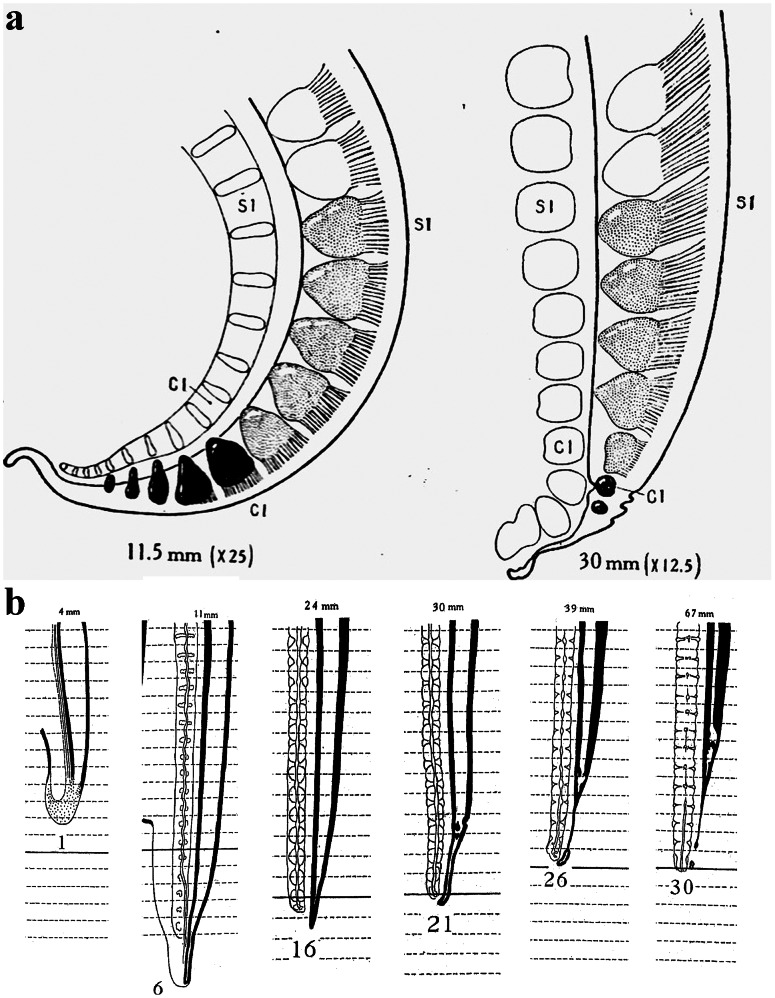
Developmental history of the FT. **a**) Illustration of two stages of a human embryo by Streeter (1919) [33], which demonstrates the de-differentiation of the caudal spinal cord into the filum terminale (FT) following the re-absorption of the vestigial tail. The numbers below the drawing indicate the length of the embryo. **b**) Drawings by Kunitomo (1918) [36]of the process of re-absorption of the embryonic tail. The numbers below the illustrations indicate the age in weeks of the fetus, and the numbers above represent the length in millimeters.

The FT is an excellent candidate as a source of autologous, multipotent cells. It provides distinct advantages over the presently available sources in that it is both easily accessible and an expendable tissue that persists in adults. It also has the potential to be used for autologous replacement therapy, thereby avoiding immunological problems. We have considered exploring the FT as a potential source of multipotent cells because of its unique developmental history [33], histologic environment [34], and propensity to produce neuroectodermal tumors [35]. Early in development, the FT provides innervation to the embryo's presumptive tail (or in rodents, temporary innervation of caudal-most tail segments). As development continues, the coccygeal/tail portion of the spinal cord gets reabsorbed and the cells undergo a process termed by Streeter as “de-differentiation” [33], [36] (Figure 1). The coccygeal spinal cord reverts to an earlier embryonic tissue type, which results in a collagenous structure with a narrow central canal that is lined by ependymal cells and surrounded by a loosely organized collection of fibroblasts, neurons, and glia [33], [36]–[38]. The resulting structure persists in adults as the FT, a slender prolongation of the caudal end of the spinal cord that anchors it to the coccyx. The postnatal FT is not interconnected with the rest of the nervous system, nor does it innervate the body. It is a vestigial remnant that is routinely sectioned in humans with a condition known as tethered cord syndrome. In these cases, the spinal cord is tightly tethered to the spine and lacks sufficient freedom of movement, so the FT is sectioned to relieve that tension [39], [40].

The histology of the FT has similarities to other CNS regions that have been previously described as NSC niches [41]. It contains peri-ventricular ependymal cells and a loosely organized collection of fibroblasts, neurons, and glia surrounding the canal [34], [38], [42]–[44]. In rats, FT neurons have been described as smaller than usual and are hypothesized to possibly represent neurons in an early stage of commitment and differentiation [45]. Paragangliomas and other primitive neuroectodermal tumors arise from the adult FT, again suggesting the possibility that NSCs are present [35], [46]–[48].

Recently, three laboratories (including our own) have identified NPCs in the human FT. In a preliminary study, Varghese *et al.* (2009) isolated neurospheres from the FT of four patients with intraspinal tumors and demonstrated that they could generate neurons and glia [49]. Arvidsson *et al.* (2011) identified NSC/NPC markers in tissue isolated from both human and rat FT. They isolated, expanded, and differentiated neurospheres from 13 out of 21 patients ranging in age from 1 to 60 years and noted a donor age-dependent decrease in proliferative potential. In addition, they isolated neurospheres from 5 out of 13 rat FTs [50]. Our laboratory has reported isolating neurospheres from the FTs of 4 fetal and 33 postnatal donors (6 months to 18 years) that were able to generate both neurons and glia. These neurospheres could also be induced to differentiate into motor neurons capable of innervating rat muscle *in vitro*. In agreement with the findings of Arvidsson *et al.*, FT tissue sections derived from 3 postnatal surgeries and from 6 autopsies (51–81 years old) stained positively for NSC/NPC markers [51].

A major difficulty in studying NPCs from the human FT is the variability of the sources. The tissue is typically obtained from tethered cord surgeries, and the donor age can vary from a few months postnatal to adulthood. The precise location of the tissue and the manner in which it is handled (including time to culture) also varies from operation to operation and naturally between clinical centers [39], [40]. Therefore, we sought to develop a rodent model for studying the FT that would permit the standardization of experiments, provide unlimited amounts of tissue, allow sampling at particular stages of the life cycle, and permit the use of transgenic animals. In this paper, we report the establishment and characterization of a rat model for generating NPCs from the FT.

## Materials and Methods

### Ethics statement

All experiments have been approved by the Institutional Animal Care and Use Committee at Harvard Medical School (protocol #04305) and were conducted in accordance with the NIH Guide for the Care and Use of Laboratory Animals.

### Culturing FT-derived neurospheres

#### Primary Culture

**Figure 2:**
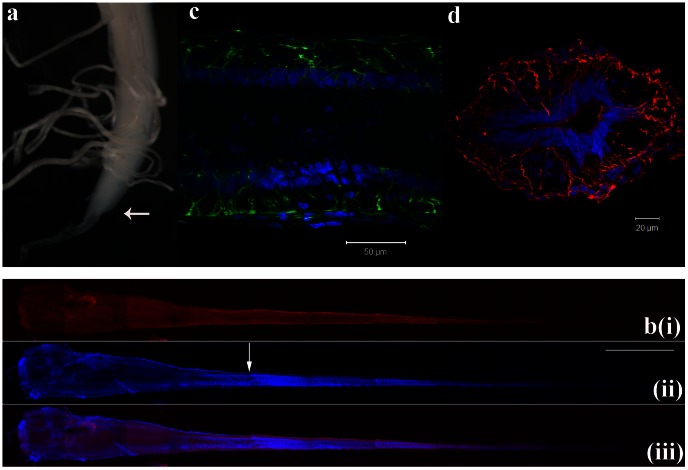
Identification of neural progenitor cell (NPC) markers in the rat FT. **a**) Dissection of a P9 FT fixed in 4% formaldehyde. The arrow indicates the start of the FT. **b**) Immunohistochemical staining of a whole mount P3 FT for the NPC marker Nestin (red, i) with the distribution of cells indicated with DAPI (blue, ii). A merged image of b(i) and (ii) is shown in b(iii). Scale bar: 1 mm. **c**) Expression of Nestin (green) and DAPI (blue) in a longitudinal section of a P1 FT. Scale bar: 50 µm. **d**) Expression of Nestin (red) and DAPI (blue) in a transverse section of a P1 FT. Scale bar: 20 µm. The arrow in b(ii) indicates the location along the FT from which this section was taken.

All experiments were conducted according to the guidelines of Harvard Medical School's Institutional Animal Care and Use Committee. Postnatal Sprague-Dawley rats (Charles River, Wilmington, MA) aged P1–P12 were anesthetized with isoflurane (Abbott, Abbott Park, IL) and killed by cervical dislocation. The vertebral column was rapidly dissected in ice-cold Hank's buffered saline solution (HBSS). The FT was visualized and identified under a microscope then dissected as illustrated in Figure 2a. Each dissection was completed in less than 5 minutes to minimize cell death. For most experiments, ∼3 FTs were pooled and transferred into a culture dish containing DMEM/F12 (1∶1), 10% fetal bovine serum (FBS), and 100 U/ml collagenase type II with 3 mM calcium. For experiments designed to determine the yield of neurospheres as a function of age, one FT was used per culture. The cultures were mechanically dissected and maintained in a humidified incubator at 37°C with 5% CO_2_. Within 15–24 hours, the tissue was rinsed, triturated, and transferred into stem cell medium (SCM; [14], [52]–[55] containing DMEM/F12 (1∶1), 1% N2 and 2% B27 supplements, EGF (20 ng/ml), and bFGF (20 ng/ml) (Invitrogen, Carlsbad, CA). The bFGF was prepared in a solution containing 8 mg/ml heparin (Sigma, St. Louis, MO) for stability. Primary stem cell proliferation was detected after 3–5 days *in vitro* and was characterized by the formation of spheres of undifferentiated cells [9].

#### Passaging Cultures

The cultures were passaged every 2–3 weeks. Neurospheres were incubated with 1X Accumax™ (Innovative Cell Technologies, San Diego, CA) for 5–8 min at 37°C followed by mechanical trituration to achieve partial dissociation. Similar results were achieved through mechanical trituration alone. Additionally, a single cell suspension could be achieved by filtering the mechanically dissociated cells through a 100 µm cell strainer (BD Falcon, Franklin Lakes, NJ). The cells were then centrifuged for 10 min at 1000 rpm and resuspended in a 1∶1 combination of fresh and conditioned medium.

### Immunocytochemistry

Whole or differentiated neurospheres were attached to glass coverslips, fixed with 4% formaldehyde in PBS (pH 7.4) for 20–30 minutes, and washed thoroughly in PBS. Primary antibodies were diluted in blocking solution (10% normal goat serum, 10% fish skin gelatin, 0.3% Triton X-100, and 0.2% bovine serum albumin in PBS), and each coverslip was incubated with the appropriate primary antibody overnight at 4°C [goat polyclonal anti-Nestin (1∶50; R&D Systems), mouse monoclonal anti-Nestin (1∶100; Millipore, Billerica, MA), mouse monoclonal Olig-2 (prediluted, Dr. Connie Cepko, HMS), mouse monoclonal anti-Vimentin (Zymed), rabbit polyclonal anti-Sox2 (1∶1000; Abcam, Cambridge, MA), rabbit polyclonal anti-β-tubulin III (Tuj-1; 1∶1000; Covance, Princeton, NJ), rabbit polyclonal anti-GFAP (1∶1000; DAKO, Glostrup, Denmark), mouse monoclonal anti-GFAP (1∶1000, Sigma), mouse monoclonal Pax6 (developed by Kawakami, A), mouse monoclonal anti-MNR2 (Developmental Studies Hybridoma Bank, NICHD, University of Iowa Department of Biological Sciences, Iowa City, IA)], goat polyclonal anti-ChAT (1∶100; Millipore), mouse monoclonal anti-MBP (1∶500, Covance), mouse monoclonal anti-neuron specific enolase (1∶1000; Millipore), and rabbit polyclonal anti-Hsp27 (1∶1000; Enzo Life Sciences, Farmingdale, NY)]. The coverslips were then washed in PBS and incubated with the appropriate secondary antibody for 4 hours [AlexaFluor488-conjugated donkey anti-rabbit, AlexaFluor488-conjugated donkey anti-mouse, AlexaFluor568-conjugated donkey anti-goat, AlexaFluor488-conjugated goat anti-mouse, and AlexaFluor568-conjugated goat anti-rabbit (all 1∶1000; Invitrogen)]. Finally, the coverslips were washed, incubated with DAPI (0.03 mg/ml) for 30 minutes, washed again, and then mounted on glass slides with Vectashield mounting media (Vector Labs). The slides were visualized for immunofluorescence using a Zeiss photomicroscope and/or with confocal microscopy. The approximate proportions of cells staining for a particular marker were determined by the average count of 4–5 20X fields that were randomly chosen by an unbiased observer. Cell counts were based on DAPI-stained nuclei.

### Rat muscle culture

Rats aged P0–P7 were sacrificed as described above, and their proximal limb muscles were rapidly dissected in ice-cold HBSS, gently teased apart, and transferred to culture dishes containing DMEM/F12 (1∶1), 1% N2 supplement, and 1% penicillin-streptomycin. Collagenase type II (100 U/ml) with 3 mM calcium was included to dissociate the muscle fibers into single cells. The dishes were incubated at 37°C with 5% CO_2_ for 24 hours, after which the cultures were triturated with a fire-polished Pasteur pipette to completely dissociate the tissue and centrifuged for 5 minutes at 1000 rpm. The pellets were washed with PBS and resuspended in medium containing DMEM/F12 (1∶1), 1% N2 supplement, 1% penicillin-streptomycin, and 10% FBS. Cis-hydroxyproline (100 µg/ml; Sigma) was added to suppress fibroblast proliferation. The cells were plated at a density of ∼10^6^ cells/ml on coverslips coated with poly-L-lysine (0.01%; Sigma) and laminin (20 µg/ml; Sigma).

### In vitro differentiation

#### Non-specific differentiation

Single neurospheres were visualized under a dissecting microscope, isolated, and plated on glass coverslips coated with poly-L-lysine (0.01%) and laminin (20 µg/ml) in individual wells of a 96-well culture dish that contained DMEM/F12 (1∶1) with 1% N2, 1% penicillin-streptomycin, and 5–10% FBS. This media was not changed for the remainder of the experiment. The coverslips were processed for immunocytochemistry either 24 hours or 7–10 days later.

#### Incubation with tritiated thymidine

Thymidine labeling experiments were conducted as previously described [56]. Briefly, neurospheres were incubated with ^3^H thymidine (NEN, 5 µCi/ml; 89 Ci/mmol) in SCM for 8 hours. The individual neurospheres were isolated, washed 3X in SCM, and differentiated in serum as described above. After differentiation, the coverslips were processed for immunocytochemistry. Prior to mounting the coverslips onto slides, emulsifier oil was added. After 2 days in the dark, the emulsifier oil was removed, and the coverslips were washed with water and then incubated in a developer for 4 minutes. Next, the developer was aspirated and the coverslips were fixed in 4% paraformaldehyde for 20 minutes. Finally, the coverslips were washed with water, mounted on slides with Vectashield mounting media (Vector Laboratories, Burlingame, CA), and visualized with fluorescence (for immunocytochemistry) or brightfield microscopy (for tritiated thymidine incorporation).

#### Directed Differentiation

Neurospheres were treated with retinoic acid (RA, 2 mM; Sigma) and either sonic hedgehog protein (Shh-N, 400–1000 nM; R&D Systems, Minneapolis, MN) or a small molecule agonist of sonic hedgehog signaling (Hh-Ag1.3, Curis, Lexington, MA) in SCM for 4–5 days using a modification of the previously described methods in Soundararajan *et al.* (2006) and Wichterle *et al.* (2002). Individual neurospheres were then isolated and plated for 7–10 days on glass coverslips coated with poly-L-ornithine (0.01%; Sigma), collagen type I (0.01%), and laminin (20 mg/ml) in individual wells of 96-well culture dishes (Corning) containing DMEM/F12 medium (1∶1) with 1% N2, 1% penicillin-streptomycin, 5% horse serum (Invitrogen), CNTF (25 ng/ml; Sigma), GDNF (25 ng/ml; Sigma), and BDNF (50 ng/ml; Sigma). Four conditions were used: (1) neurospheres treated as above; (2) as above but without RA; (3) as above without Shh or RA; (4) serum alone (without Shh, RA, or the 3 neurotrophins). The coverslips were subsequently processed for immunocytochemistry.

#### Neuromuscular junction formation

Individual neurospheres were treated with RA (2 mM) and Shh-N (400–1000 nM) for 4–6 days and subsequently plated on muscle cultures in the differentiation media for motor neuron growth and survival described above. We used two types of control cultures: 1) myocytes alone and 2) myocytes onto which untreated neurospheres were plated. After 6–21 days, the cultures were incubated with AlexaFluor594-conjugated-α-bungarotoxin (2 µg/ml; Invitrogen) for 2.5 hours, washed, fixed, and processed for immunocytochemistry. Single neurospheres were labeled for 1 hour prior to co-culture with either 1 µM Di-I, Di-D, or carboxyfluorescein diacetate succinimidyl ester (CFSE).

### In vivo transplantation of FT-derived NPCs

#### Isolation of eGFP+ neurospheres

Transgenic rats expressing enhanced GFP under the chicken β-actin promoter and cytomegalovirus enhancer (“green rat” CZ-004, SD-Tg(Act-EGFP)CZ-004Osb; SLC, Shizuoka, Japan) were obtained from Dr. Kocsis at Yale University. Primary cultures of neurospheres were established using the same protocol described above for wildtype rats.

#### Transplantation of FT-derived NPCs into chick embryos

GFP-expressing neurospheres were grown as described above. After 4 ml of albumin had been removed to accommodate the transplanted tissue, either whole neurospheres or dissociated NPCs were implanted into a stage 10–17 chick embryo at the rostral end of the developing spinal cord using a pulled class pipette and microinjector apparatus. A saline control was used to mimic NPC injection, and in some cases, 0.1% Fast Green was co-injected to add contrast. The embryos were then placed horizontally in a 38°C incubator until the tissue was harvested at 3–7 days post-transplantation, fixed with 4% PFA, sectioned at 20–50 µm, and labeled with appropriate antibodies to assess the differentiation potential of the implanted NPCs.

## Results

### Characterization of the FT in vivo

The FT was isolated from rats aged P1–P12 (n = 63) by excising the tissue at the caudal end of the spinal cord that extends past the conus medullaris (Figure 2a). To verify the presence of NPCs in the FT, a whole mount FT underwent immunohistochemical staining for the NPC marker Nestin (tiled confocal images are shown in Figure 2b(i)). Co-staining with the nuclear marker DAPI indicates the overall distribution of cells throughout the FT (Figure 2b(ii)). This result suggests that Nestin-positive cells are present through the entire length of the FT (n = 14). To examine the distribution of these Nestin-positive cells more closely, sections were cut from the first third of the FT closest to the conus medullaris (indicated by the arrow in Figure 2b(ii)). The pattern of Nestin staining in longitudinal sections (n = 5) suggests that the Nestin-positive cells possess long processes that extend from the central canal to the pial surface, while their cell bodies reside within or close to the ependymal zone (Figure 2c). The distribution of Nestin-positive cells in transverse sections at the same location along the length of the FT (n = 9) confirmed their morphology and distribution (Figure 2d). These results suggest that NPCs are indeed present in the FT.

### Isolation and characterization of self-renewing FT-derived neurospheres

#### Isolation

**Figure 3:**
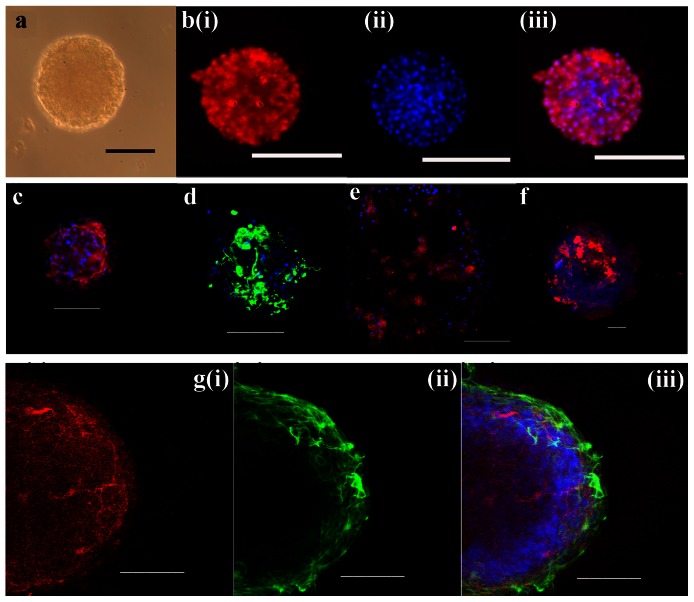
Characterization of FT-derived, undifferentiated neurospheres by the expression of NPC, neuronal, and glial markers. **a**) Phase microscopy of a single neurosphere derived from a P10 FT culture after 10 days *in vitro* (DIV) and 1 passage. **b**) An individual neurosphere derived from a P5 FT culture (5 DIV) stained for the NPC marker Nestin (i, red), DAPI (ii, blue), and merged (iii). The images in (c–f) are co-stained with DAPI (blue). **c**) The NPC marker Olig2 (red) is expressed in a neurosphere derived from a P7 FT culture (34 DIV). **d**) Expression of Vimentin (green) in a proportion of cells of an individual neurosphere derived from a P7 FT culture (60 DIV). **e**) Immunostaining of Sox2 (red) in some cells from a single neurosphere isolated from a P6 FT culture (30 DIV). **f**) Weak staining of Musashi (red), with some hot-spots, in a neurosphere isolated from a P6 FT culture (30 DIV). **g**) (**i**) Sparse expression of β-Tubulin III (Tuj-1) in a proportion of cells of a neurosphere derived from a P7 FT culture (34 DIV). The expression of GFAP in the same neurosphere (**g-ii**) is restricted to the peripheral cells *in vitro*. (**iii**) Merged images of g(i) and (ii). Scale bars: 100 µm.

Cells isolated from the FT were dissociated with collagenase and cultured in standard stem cell medium (DMEM/F12+1% N2 supplement) containing bFGF (20 ng/ml) and EGF (20 ng/ml) along with B27 (2%) and N2 (1%) supplements. Previous studies have identified these mitogenic factors as successful stimulants of NPC proliferation [14], [53], [57]. Each culture was derived from a single donor rat (ages P4–P10). After 3–4 days *in vitro*, neurospheres were observed in 96 out of the 100 primary cultures. These neurospheres were primarily free-floating and were identified by their spherical structure, phase bright appearance, and regular cell membranes (Figure 3a). The neurospheres initially appeared as smaller clusters of 3–4 round cells and eventually grew into larger neurospheres. The size of these larger neurospheres ranged widely from <50 µm to >1 mm. Cell clusters of <30 µm were not counted as neurospheres [58]. The number of neurospheres per primary culture varied from 1 to more than 40. For a subset of the cultures, we examined the effect of donor age upon the ability to produce neurospheres and the number of neurospheres obtained and found no correlation with age (Table 1). To demonstrate their capacity for proliferation and self-renewal, neurospheres were dissociated and passaged up to 19 times. These cultures have been maintained *in vitro* for up to 7 months. Twelve cultures have been frozen, and two have been tested for viability and were successfully recovered upon thawing.

**Table 1:** Lack of correlation between donor age and culture success/number of FT-derived neurospheres.

Donor Age (days)	Fraction of cultures producing neurospheres	Average number and range of neurospheres
P4	23/23	7.6; (2–18)
P5	21/22	10.8; (0–42)
P6	18/18	8.7; (1–31)
P9	3/3	6.7; (6–8)

A potential effect of donor age upon the ability to produce neurospheres and the number of neurospheres obtained was systematically examined on a subset of cultures. Donor rats of all ages had a similar culture success rate, and similar numbers of neurospheres were obtained for all ages.

#### Characterization

Contrary to earlier thinking, neurospheres are not homogenous populations of cells, but have instead been shown to be a heterogeneous collection of NSCs and NPCs that likely have varying differentiation potentials [53], [59], [60]. We characterized the FT-derived neurospheres using immunocytochemistry to determine the expression of various NSC, NPC, neuronal, and glial markers. Specifically, neurospheres were stained for the NSC/NPC markers Nestin (n = 9), Sox2 (n = 8), Vimentin (n = 6), Olig-2 (n = 3), and Musashi (n = 4), the neuron-specific marker β-tubulin III (Tuj-1, n = 12), and the astrocyte marker glial fibrillary acidic protein (GFAP, n = 12).

In all cases, a varying proportion of cells were positive for Nestin (Figure 3b). In 4/9 cases, 100% of the cells in the neurosphere were Nestin^+^, and this did not appear to be correlated with the amount of time in culture. Staining for other neural progenitor markers was variable. In 3/3 experiments, 100% of cells within the neurosphere stained positive for Olig-2 with some areas showing more intense staining (Figure 3c). Although all neurospheres had some proportion of cells that stained positive for Vimentin and Sox2, (Figures 3d,e, respectively), this percentage varied from 33–100% for Vimentin and 40–100% for Sox-2. Musashi staining was weak, with occasional clusters of high intensity staining (Figure 3f).

Tuj-1^+^ and GFAP^+^ cells were present in all neurospheres (Figure 3g; n = 12). Every neurosphere contained some cells that were positive for both markers, and this fraction varied greatly among individual neurospheres. As shown in Figure 3g, there was spatial clustering of cells expressing the different markers. While this clustering was apparent in most neurospheres, the patterns were variable.

### Differentiation of FT-derived neurospheres into neurons and glia

**Figure 4:**
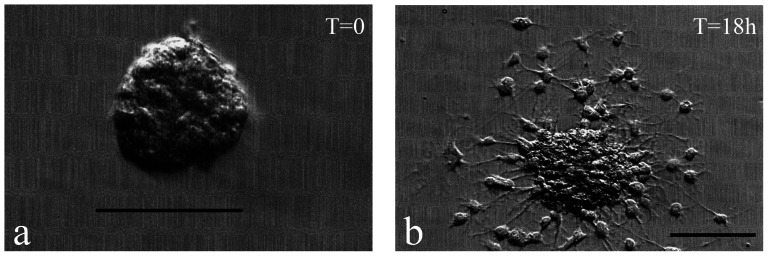
Inducing differentiation of FT-derived neurospheres. **a**) An **i**ndividual neurosphere from a P5 FT (42 DIV) was plated at T = 0 on a laminin-coated coverslip and cultured in media that contained 10% serum. **b**) Morphological properties of differentiation were evident after 18 hours of exposure to these differentiating conditions. Scale bar: 100 µm.

Some neurospheres adhered to the cultureware and spontaneously differentiated into cells having the morphological characteristics of neurons and glia without the addition or removal of factors from the medium. We sought to determine the conditions required to differentiate FT-derived neurospheres into neurons and glia. After withdrawing bFGF and EGF, single neurospheres were plated onto coverslips that had been treated with 7 different combinations of adhesive substrates ± exposure to 5–10% fetal bovine serum (Table 2). For each condition, 5 experiments were performed. After 7 days, the cultures were stained for Tuj-1, neurofilament, O1, GFAP, and Nestin. Although the use of either an adhesive substrate alone or serum alone was sufficient to initiate morphological differentiation, the addition of serum resulted in a more rapid differentiation. For example, an individual neurosphere plated on a poly-L-lysine- and laminin-coated coverslip began to exhibit morphological properties of differentiation after 42 hours (data not shown). In contrast, a neurosphere plated on a laminin-coated coverslip and cultured in 10% serum-containing medium began to differentiate after only 18 hours (Figures 4a,b). In all cases, we detected cells derived from the neurospheres that expressed either neuronal or glial markers including Tuj-1, neurofilament, O1, and GFAP (data not shown).

**Table 2:** Conditions for FT-derived neurosphere differentiation.

Immunocytochemical marker					
Differentiating Conditions	O1	GFAP	BTIII	Neurofilament	Nestin
Polylysine	+	+	+	+	−
Laminin	+	+	+	N.D	−
Serum	+	+	+	+	−
Polylysine+laminin	+	+	+	+	−
Polylysine+serum	+	+	+	+	−
Laminin+serum	+	+	+	+	−
Polylysine+laminin+serum	+	+	+	+	−

Seven different conditions involving various combinations of an adhesive substrate with serum-containing medium were used to differentiate FT-derived neurospheres. After 7 days, the differentiated cells were subsequently immunostained for markers to identify neurons (βTIII, neurofilament), astrocytes (GFAP), and oligodendrocytes (O1). Although all seven conditions generated these cell types, exposing neurospheres to serum resulted in a more rapid differentiation process. N.D.: not done.

All subsequent differentiation experiments (n = 65) were conducted by withdrawing the growth factors, supplementing the media with 5–10% fetal bovine serum, and plating single neurospheres onto coverslips coated with both poly-L-lysine and laminin. The cells were cultured under these conditions for 24 hours (n = 30) or 7–10 days (n = 35), and the cultures were subsequently fixed for immunocytochemistry. Given the wide distribution of neurosphere sizes used in these experiments, the number of differentiated cells obtained ranged from <50 to >5000 cells per neurosphere, which correlated with the size of the neurosphere that had been plated. Larger neurospheres (usually >100 µm) were capable of generating >5000 differentiated cells. In all cases, the cells began to migrate away from the original neurosphere and develop extensive processes within 18 hours of exposure to the differentiation conditions (Figure 4).

**Figure 5:**
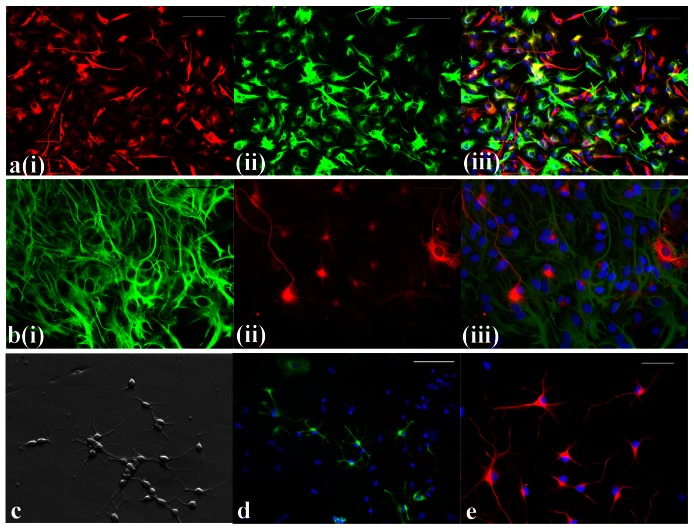
Differentiation of FT-derived neurospheres into neurons, astrocytes, and oligodendrocytes. **a**) Differentiated cells derived from a single neurosphere double stained for the NPC marker Vimentin (red, a(i)), the astrocyte marker GFAP (green, a(ii)), and merged and co-stained with DAPI (a(iii)). These cells were derived from a single neurosphere that was plated on a poly-L-lysine- and laminin-coated coverslip and grown in 10% serum-containing medium without EGF, bFGF, and LIF for 7 days prior to immunocytochemistry. Donor: P4 FT (15 DIV) after 1 passage. **b**) Differentiated neurons and glia from an individual neurosphere double stained for the astrocyte marker GFAP (green, b-i) and the neuronal marker Tuj-1 (red, b-ii). The merged image co-stained with DAPI is shown in **b-iii.** Same conditions as (a). Donor: P7 FT, 30 DIV. **c**) Phase image of differentiated cells from a FT-derived neurosphere. **d**) Cells differentiated from a single neurosphere, stained for the oligodendrocyte marker O1 (green) and DAPI (blue). Same conditions as (a). Donor: P6 FT, 36 DIV. **e**) Cells derived from a single neurosphere and cultured in the same differentiating conditions as (a) were stained for the astrocyte marker GFAP (red) and DAPI (blue). Donor: P7 FT, 30 DIV. Scale bars: a&c, 100 µm; b&d, 50 µm.

We next examined whether the neurospheres were capable of producing NPCs, neurons, oligodendrocytes, and astrocytes by assessing their expression of the NPC marker Vimentin (Figure 5a), the neuronal marker Tuj-1 (Figure 5b), the oligodendrocyte marker O1 (Figure 5d), and the astrocyte marker GFAP (Figure 5e). In most cases, we double stained the cells derived from a single neurosphere for two of these markers (Figures 5a,b). FT-derived neurospheres had varied differentiation potentials; however, we consistently observed neurons, oligodendrocytes, and astrocytes in each experiment (n = 12). In addition to their immunocytochemical profiles, Figure 5 also illustrates that the differentiated cells displayed morphologies characteristic of neurons, oligodendrocytes, and astrocytes.

To confirm that the differentiated cells were derived from proliferative cells, we labeled actively dividing cells with tritiated thymidine [51]. Neurospheres were treated with ^3^H for 8 hours (n = 5). They were then washed and differentiated over 7 days in the standard conditions described above. Derived cells were stained for Tuj-1 and GFAP. In all 5 cases, 27–90% of the cells identified immunologically as neurons and glia had incorporated tritiated thymidine into their nuclei (data not shown). This demonstrates that the derived cells were the progeny of actively dividing cells.

**Figure 6:**
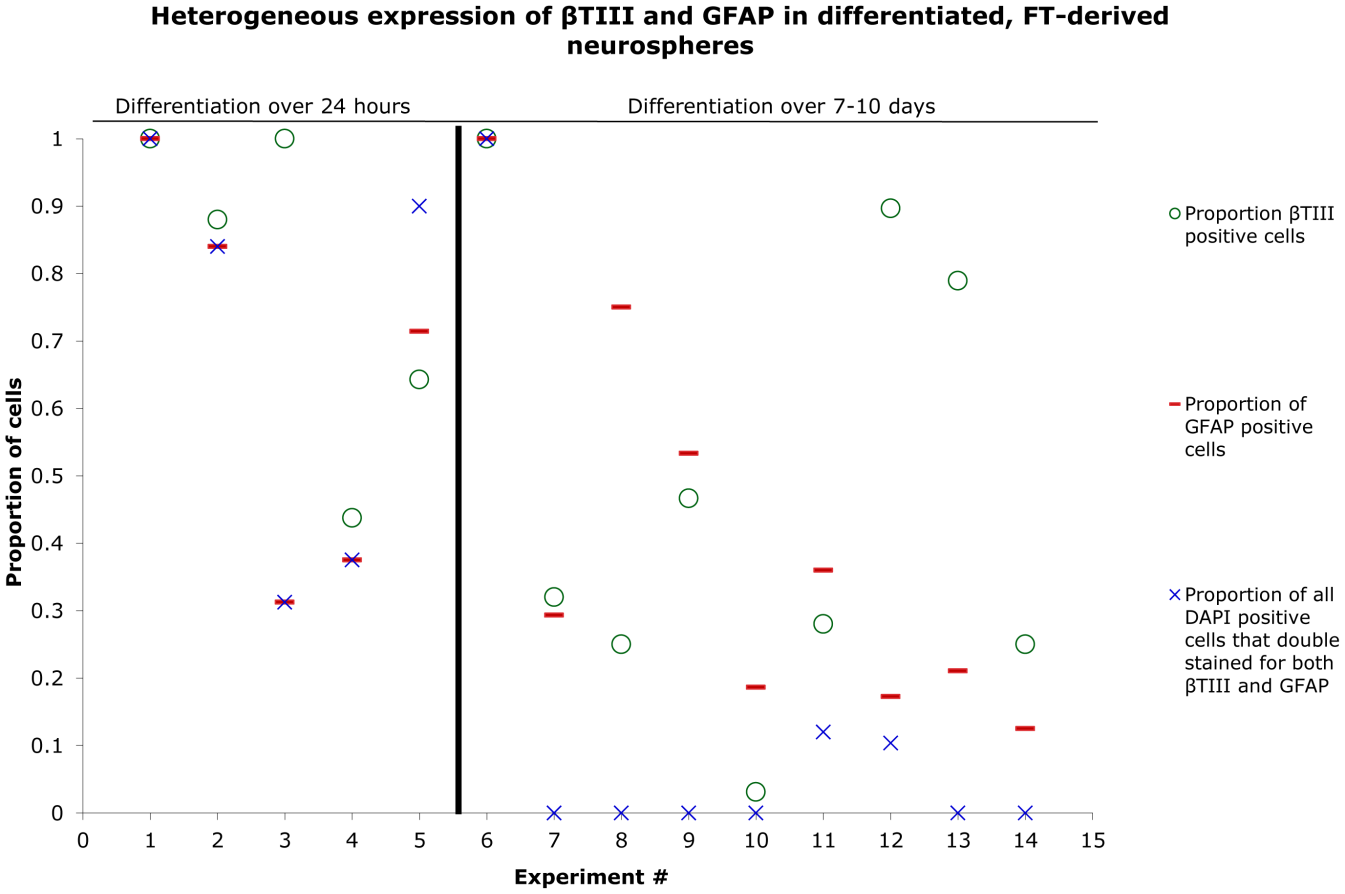
Heterogeneous differentiation potential of FT-derived neurospheres. This scatter graph illustrates the variability in the expression of Tuj-1 (neuronal marker) and GFAP (astrocytic marker) in 14 neurosphere differentiation experiments. Individual neurospheres were differentiated by both attachment to an adhesive substrate (poly-L-lysine- and laminin-coated coverslips) and exposure to 5% serum. Differentiated cells were evaluated by immunocytochemistry after either 24 hours or 7–10 days. In both cases, a variable proportion of neurons and astrocytes was observed. The approximate proportion of differentiated cells from a neurosphere that co-stained for both markers decreased after 7–10 days of exposure to the differentiating conditions relative to the proportion of overlap noted after 24 hours.

Figure 6 illustrates the variable expression of Tuj-1 and GFAP in 14 experiments comparing differentiation after 24 hours to differentiation after 7–10 days. Neurospheres differentiated over 24 hours (n = 9) resulted in a high proportion of cells that double stained for both neuronal and glial markers (Figure 6). In the case of Tuj-1 and GFAP staining, 69±14% (n = 5) of the cells were double stained for the two markers. After 7–10 days, the proportion of cells that double stained for both neuronal and glial markers decreased significantly (Figure 6). In the case of Tuj-1 and GFAP staining, only 13±10% (n = 9) of the cells were double stained for the two markers (Figures 5b, 6). On rare occasions, after 7 days of differentiation, >85% of cells derived from a single neurosphere expressed *either* a neuronal or glial marker (n = 2). However, in most cases, no obvious predominance was observed, and varying proportions of both neuronal and glial cells were noted from the differentiation of a single neurosphere (Figures 5,6). These varying proportions of Tuj-1^+^ and GFAP^+^ may reflect the heterogeneous differentiation potential of each neurosphere. This variation persisted in neurospheres either from the same source or different sources regardless of the age of the rat.

### FT-derived neurospheres can be directed to generate motor neurons (MNs)

**Figure 7:**
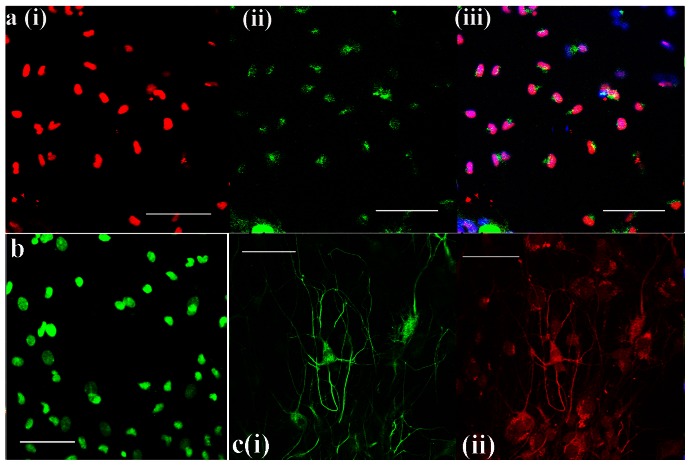
Generation of motor neurons (MNs) from FT-derived neurospheres. Individual neurospheres were treated with RA and either Shh-N or Hh-Ag1.3 for 4–5 days, plated onto an adhesive substrate, and cultured in serum-containing medium with appropriate growth factors for 7–10 days. Differentiated cells were subsequently evaluated for the expression of MN and MN progenitor markers. **a**) Differentiated cells from a P7 FT (30 DIV) stained positive for Olig2 (i, red) Pax6 (ii, green). The merged images co-stained for DAPI (blue) are shown in (iii). **b**) The MN-specific marker MNR2 is expressed by FT-derived neurospheres (green). Donor: P7 FT, 30 DIV. **c**) Expression of Tuj-1 (i, green) and ChAT (ii, red). The merged image co-stained for DAPI is shown in (iii). Donor: P6 FT, 36 DIV. Scale bars: 100 µm.

We next determined whether FT-derived neurospheres were capable of generating spinal cord MNs that could be used in cell replacement strategies in cases of spinal cord trauma or MN degeneration. To produce MN progenitors, single neurospheres were treated for 4–5 days with 2 µM retinoic acid and either 0.5–1 µM sonic hedgehog protein (Shh-N) or 1.5 µM of the hedgehog agonist Hh-Ag1.3. The neurospheres were subsequently plated on an adhesive substrate in the presence of 5% horse serum and 3 neurotrophic factors known to promote MN growth and survival (ciliary-derived neurotrophic factor (CNTF), brain-derived neurotrophic factor (BDNF), and glia-derived neurotrophic factor (GDNF); Soundararajan *et al.*, 2006 and Wichterle *et al.*, 2002). In all experiments (n = 25), various proportions of the differentiated cells expressed either MN progenitor or mature MN markers (Figure 7). At first, various proportions of differentiated cells expressed motor neuron progenitor markers including Olig2 and Pax6 (Figure 7a). After 7–10 days, the fraction of MNs produced by each neurosphere was determined using immunocytochemistry for the MN marker Motor Neuron Restricted-2 (MNR-2; Figure 7b). Motor neurons were further characterized by their expression of the neuronal marker Tuj-1 as well as choline acetyl transferase (ChAT), which is the enzyme necessary for the synthesis of acetylcholine, the neurotransmitter used by motor neurons (Figure 7c).

**Figure 8:**
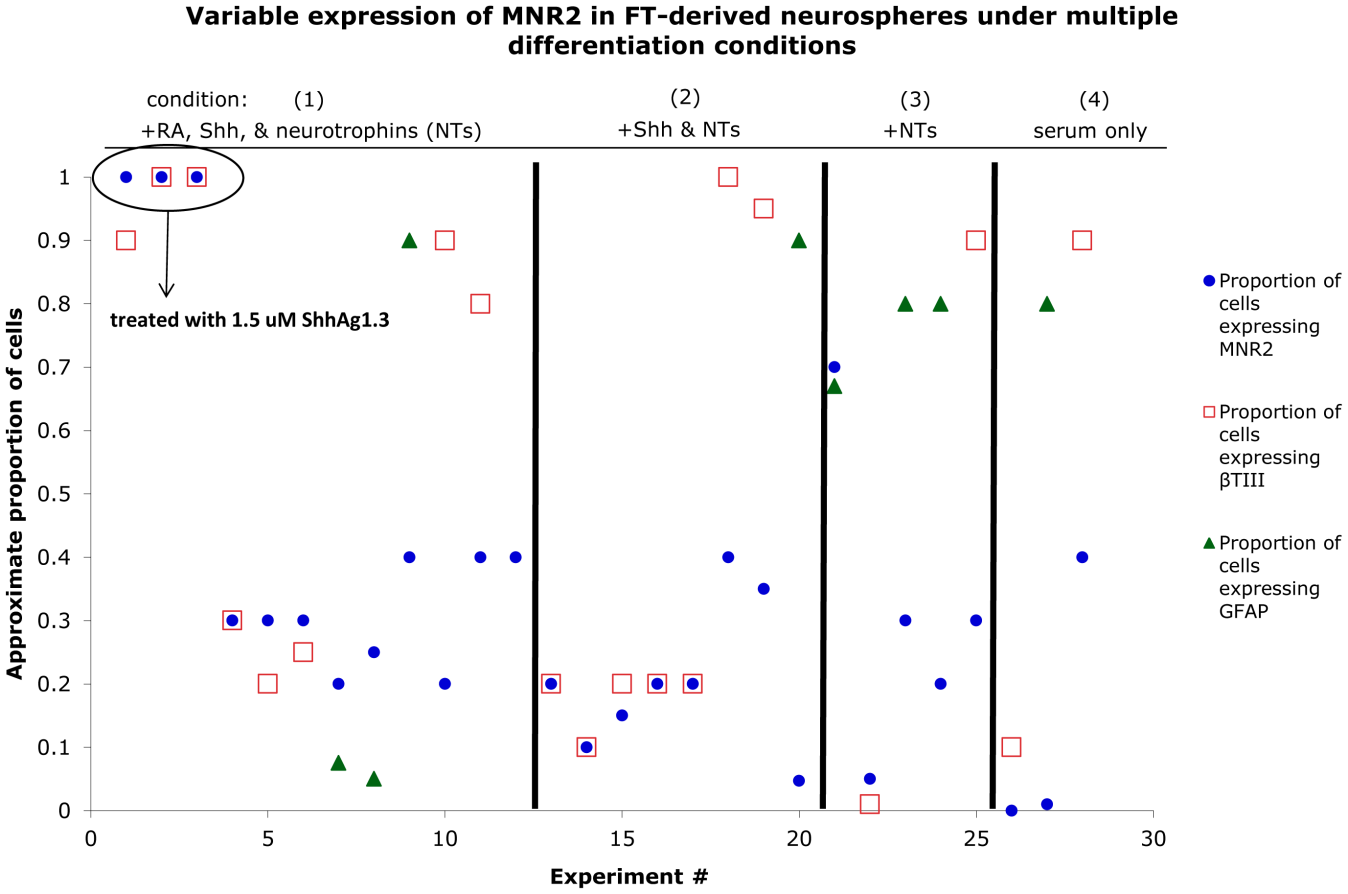
Variable expression of MNR2 in FT-derived neurospheres undergoing MN differentiation. Scatter graph illustrating the variability in MNR2 expression in differentiated neurospheres under four sets of differentiation conditions. The X axis is the experiment number, and the Y axis is the average proportion of cells derived from each neurosphere expressing MNR2, Tuj-1 and/or GFAP. MNR2, which was used to identify MN generation, was expressed by cells in roughly the same proportion among conditions (1), (2), and (3) with more inconsistent results under condition (4). In 3 of the 28 experiments where the small molecule agonist, Hh-Ag1.3, was used in place of Shh-N, 100% expression of MNR2 was observed. See Results for more detail.

While neurospheres treated with Shh-N gave rise to differentiated neurons, only 20–40% of these neurons expressed MN markers such as MNR2, Isl1, Lim3 and ChAT (n = 14). Increasing the Shh-N concentration from 400 to 1000 nM did not appear to alter the outcome. When we instead used the hedgehog agonist Hh-Ag1.3 (n = 9, 1.5 µM), 95–100% of the differentiated neurons expressed MN markers (Figure 8). This suggests that at these concentrations, the agonist may be more effective than the actual peptide in generating MNs from FT-derived neurospheres.

We also varied the differentiation conditions to determine which factors were essential for generating MNs from the FT. The following conditions were used: (1) neurospheres were treated as described above (with RA and Shh) and then differentiated in media containing serum along with BDNF, CNTF, and GDNF (n = 25); (2) neurospheres were treated with Shh-N but without RA (n = 8) followed by the addition of serum and the three neurotrophins; (3) untreated neurospheres were cultured in media containing serum and the three neurotrophins (n = 8); and (4) untreated neurospheres were differentiated in media containing serum without the addition of neurotrophic factors (n = 3). Under conditions (2) and (3), neurospheres consistently generated a variable proportion of MNR2^+^ cells (5–67%). In condition (4), the generation of MNs was inconsistent (Figure 8). In 1/3 of the cases, 40% of cells derived from the neurosphere expressed MNR2, and in 2/3 cases no cells were MNR2^+^. The use of RA and Shh-N for directed and consistent generation of MNs did not prove superior to simply differentiating the neurospheres in the presence of BDNF, CNTF, and GDNF. However, Hh-Ag1.3 seems to be beneficial in increasing the MN yield (Figure 8). Given that FT is the vestigial remnant of the spinal cord, these results may indicate an innate potential of some FT NPCs to differentiate into MNs without either the caudalizing action of RA or exogenous ventralizing Shh signaling.

### Muscle co-culture and neuromuscular junction (NMJ) formation

**Figure 9:**
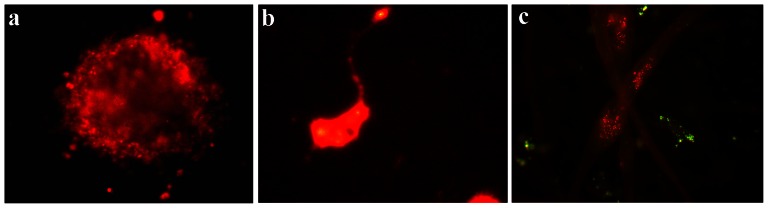
Neuromuscular junction formation. **a**) A single neurosphere derived from a P6 FT was treated for 24 hours with Shh and RA and then labeled with DiI. **b**) A cell derived from the DiI-labeled neurosphere that has been co-cultured with rat myocytes for 2 days. **c**) After 20 days of co-culture, muscle cells were labeled with α-bungarotoxin-AF594 (red) to identify acetylcholine receptors clustered at neuromuscular junctions. Cells derived from individual neurospheres were pre-labeled with CFSE (green) prior to being added to the co-culture.

To assess the potential of FT-derived MNs to innervate muscle, we used the same motor neuron/muscle co-culture system that we had previously developed for human FT-derived NPCs [51]. Single neurospheres were treated with RA & Shh and co-cultured with postnatal rat striated muscle fibers under differentiating conditions in the presence of BDNF, CNTF, and GDNF (n = 24). To confirm that the neurons observed in the co-culture were derived from plated neurospheres, 14 neurospheres were pre-incubated with either a lipophilic carbocyanine dye (Di-I or DI-D; Figure 9a,b) or carboxyfluorescein diacetate succinimidyl ester (CFSE; Figure 9c). Treated neurospheres were co-cultured with muscle for 6–21 days and then incubated with fluorescent α-bungarotoxin to mark the nicotinic acetylcholine receptors and identify the presence of neuromuscular junctions. After 6–21 days, clustered α-bungarotoxin staining on muscle fibers was detected in all of the co-cultures (Figure 9c). In contrast, control cultures containing muscle fibers only do not contain neuromuscular junctions as defined by acetylcholine receptor clustering [51]. These findings are highly suggestive that FT-derived neurospheres have the potential to form neuromuscular junctions.

### FT-derived neurospheres can differentiate into neurons and glia *in vivo*

**Figure 10:**
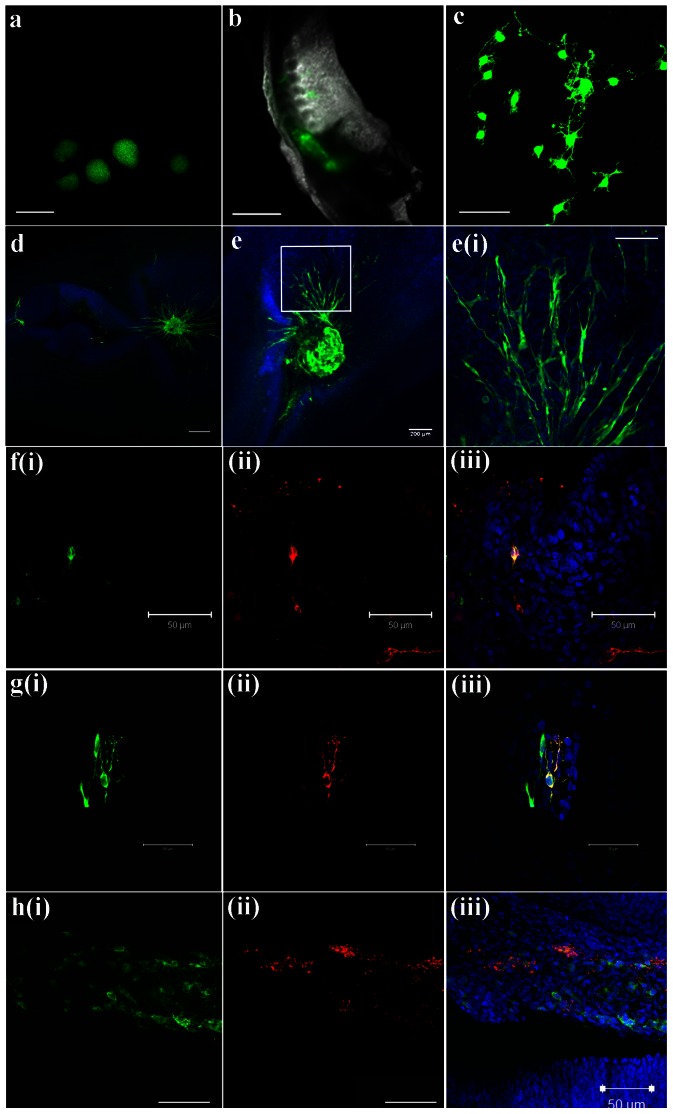
*In vivo* survival and differentiation of FT-derived NPCs transplanted into the developing chick spinal cord. **a**) Neurospheres were isolated from a P2 transgenic rat that expressed eGFP in every cell. Scale bar: 100 µm. **b**) GFP^+^ NPCs from a P2 rat were transplanted into a stage 10 (33 hrs) chick neural tube at the prospective rostral end of the developing spinal cord. This image was taken immediately after injection, and the rostral end is pointing towards the bottom. Scale bar: 200 µm. **c**) After 3 DIO (∼stage 22), GFP^+^ cells had survived and begun to take on the morphology of neurons and glia. Scale bar: 50 µm. **d–e**) Neurospheres from a 6-month-old human were transplanted into a stage 10 (33 hrs) chick spinal cord at the rostral end. After 3 DIO, transplanted cells were labeled with the human-specific antibody HSP27 (green) and can be seen migrating away from the transplantation site. Two different embryos are shown in (d) and (e). The image in (e)i is a magnified view of the migrating cells from the highlighted area in (e). Scale bars: (d) and (e): 200 µm, (e)i: 50 µm. **f–h**) After 7 DIO, some FT-derived NPCs (green, f–h(i)) expressed the neuronal marker Tuj-1 (red, f(ii)), the astrocyte marker GFAP (red, g(ii)), or the oligodendrocyte marker MBP (red, h(ii)). In all cases (f–h), a merged image of (i) and (ii) along with the nuclear marker DAPI is shown in (iii). Scale bars: 50 µm.

Because FT-derived neurospheres could differentiate into both neurons (including motor neurons) and glia *in vitro*, we wanted to examine the behavior of these cells when they were reintroduced to the spinal cord *in vivo*. To accomplish this, the FT was isolated from transgenic rats aged P0–P2 in which all cells express GFP under the control of the chicken β-actin promoter and cytomegalovirus enhancer (SD-Tg(Act-EGFP)CZ-004Osb; SLC Japan). Neurospheres were isolated and cultured from this tissue as previously described (Figure 10a). These FT-derived, GFP+ neurospheres were implanted into the neural tube of stage 10–15 chick embryos at the rostral end of the developing spinal cord (Figure 10b; n = 31). In some cases, the neurospheres were dissociated before being implanted. The embryos were then permitted to develop for an additional 3–7 days post-transplantation before their ability to survive, migrate into the host tissue, and differentiate was assessed.

An analysis of the spinal cord from these operated chick embryos 3 days post-implantation revealed the presence of a number of GFP+ cells that had not only survived but had migrated away from the injection site into the developing chick spinal cord (n = 27). These cells clearly had the morphology of neurons and glia (Figure 10c). After 7 days, immunohistochemical analysis revealed that a subset of these transplanted GFP^+^ cells could be identified as neurons (Figure 10f; n = 12), astrocytes (Figure 10g; n = 9), and oligodendrocytes (Figure 10 h; n = 9) by their expression of the immunohistochemical markers Tuj-1, GFAP, and myelin basic protein (MBP), respectively. In addition, we also implanted FT-derived cells from human donors aged 3 months to 2 years into the developing chick spinal cord (n = 8; see [51] for details on how human FT-derived NPCs were obtained). These human NPCs were also able survive, migrate away from the injection site, and differentiate into both neurons and glia over the course of 3–5 days (n = 5; Figure 10d,e). This preliminary result suggests that rat and human FT-derived NPCs have the ability to differentiate into both neurons and glia *in vivo*. We have not yet been able to follow the chicks' development for a sufficient period of time to determine whether these cells have the ability to integrate into the host nervous system.

## Discussion

The results of this study demonstrate that multipotent stem/progenitor cells are present in the postnatal FT. These cells exhibit two cardinal properties of NPCs: they are capable of both self-renewal/expansion and of differentiation into multiple cell types, including neurons, astrocytes, and oligodendrocytes [61]. The results described here are consistent with the findings of three previous studies including one from our own lab, which have all demonstrated that neural stem/progenitor cells can be derived from the postnatal human FT [49]–[51]. One of these reports had suggested that the rat FT was also capable of producing NPCs; however, the observed efficiency in that study was only 39% [50]. Here, we have optimized the rat neurosphere assay to achieve a 96% culture success rate at a range of donor ages. Presumably, the improved efficiency is due to this being an extended study involving large numbers of rats. In addition, we have demonstrated that FT-derived NPC cells can be differentiated into MNs. The ability of the FT to generate MNs may be of particular therapeutic significance for neurodegenerative diseases such as amyotrophic lateral sclerosis (ALS). The cells were not assessed for clonality because this is not immediately applicable therapeutically [2], [3]. We reasoned that so long as FT-derived NPCs can be produced in large enough numbers to permit cell replacement strategies, clonality is not essential to the therapeutic strategy.

### The neurosphere assay

We employed the neurosphere assay for isolating and expanding NPCs. As observed in this study, individual neurospheres demonstrate the two classic NPC properties: self-renewal and multipotency, which suggests that the FT-derived cells are in fact neural progenitors. For the past 20 years, the most widely used technique for culturing NPCs has been to utilize non-adherent conditions under which these cells aggregate and form spheres of proliferating cells called neurospheres [9], [62]. However, recent studies have raised some issues regarding the limitations of the neurosphere assay. One concern has been that a single neurosphere may contain a heterogeneous population of cells, and culture conditions may favor the growth and survival of one type of cell over another. In fact, each neurosphere may contain only a small number of NPCs that would then become diluted with each passage [62]. One non-neuronal cell type likely to be present initially in the neurospheres is ependymal cells, which line the central canal of the FT. However, these cells are postmitotic [63] and are diluted out with each passage; therefore, they are unlikely to contribute to the population of dividing cells observed in this study. Additionally, the neurosphere assay may not take into account quiescent cells that are residing in G0 at the time of neurosphere formation [64]. Cell density is another concern because as the neurosphere grows, it becomes difficult for nutrients to reach the innermost cells, and a necrotic center may begin to form [65].

Thus, some groups instead prefer to utilize a monolayer assay where NPCs are grown under adherent conditions to form a single layer of cells. This results in a more homogeneous population of cells and reduces the level of spontaneous differentiation among the NPCs [64]. Recently, we have started culturing FT-derived NPCs under monolayer-forming conditions with encouraging success, and we intend to employ this method going forward.

### Cell identity manifests after 7–10 days in differentiating conditions but may be determined prior to the differentiation process

We compared neuronal and glial marker expression in single neurospheres that had been differentiated for 24 hours versus 7–10 days. After 24 hours, most cells expressed both sets of markers, which may reflect an unresolved cell fate early on in the differentiation process. In contrast, after 7–10 days, most cells derived from a single neurosphere expressed either a neuronal or a glial marker with very few cells double staining for both. This did not vary with donor age and may be the result of cells expressing a more committed cell fate over an extended period of time compared with a relatively ambiguous cell identity after 24 hours.

Cell identity may also be determined prior to the differentiation process. Before differentiation, neurospheres expressed different patterns of Tuj-1 and GFAP staining despite identical treatment. While some cells within a neurosphere indicated double staining for both markers, most expressed only a single marker. Cells positive for the same marker tended to cluster together spatially. This is consistent with reports of heterogeneous neurospheres that have been derived from other adult CNS and embryonic sources [59], [60], [66]. Suslov *et al.* (2002) studied the cDNA libraries of 30 neurosphere clones and found that they all differentially expressed transcripts. This group also demonstrated that neurospheres contained NSCs as well as neuronal and glial progenitors in different stages of differentiation with distinct neural developmental commitments [60].

It is possible that the neuronal or glial characteristics acquired prior to neurosphere differentiation may predict the differentiation potential of each neurosphere. Temporary double staining during early stages of differentiation may represent a point along the differentiation pathway where cell fate remains ambiguous. Early staining patterns among neurosphere cells (GFAP^+^ or Tuj-1^+^) before differentiation potentially implies that manipulating neurospheres towards a more neuronal or glial fate may require culturing them in different conditions from the outset.

### Neural progenitor cell (NPC) markers persist at 7–10 days

NPC marker expression was high after 24 hours of differentiation and even higher after 7–10 days. This was surprising because most cells by this point begin to express markers representing a more mature phenotype, i.e., a neuronal or glial cell marker. Cells that persist in expressing NPC markers in addition to neuronal- or glial-specific markers may be lineage-specific NPCs. Alternatively, this persistence and/or increase in NPC marker staining may be a tissue culture artifact.

### FT-derived neurospheres have an innate potential to generate MNs

FT-derived neurospheres generated MNs with and without exposure to RA and Shh, which have both been previously used to differentiate embryonic stem cells into MNs *in vitro* [67]–[69]. Rostral neural progenitors in embryoid bodies acquire a spinal positional identity in response to RA (a caudalizing signal) and subsequently attain a motor neuron progenitor identity in response to the ventralizing signals of Shh [69]–[72]. BDNF, CNTF, and GDNF are neurotrophins that are known to support MN growth and survival [73]–[76]. FT-derived neurospheres that were treated with RA and Shh-N prior to differentiation in media containing serum, BDNF, CNTF, and GDNF generated 20–40% MNs. Neurospheres plated in serum with BDNF, CNTF, and GDNF without RA or Shh-N treatment generated between 5–67% MNs, which suggests that treating FT-derived neurospheres with Shh-N and RA is not more effective in generating MNs than plating them in the presence of BDNF, CNTF, and GDNF alone. While RA and Shh are crucial in directing embryonic stem cell differentiation into MNs, they may not be as critical to a subset of FT-derived NPCs, which have possibly been caudalized and ventralized during embryonic development. When FT derived neurospheres were plated in serum alone, 1/3 generated 40% MNs, **i**ndicating that occasional MN differentiation can occur without addition of RA/Shh-N or growth factors. This may be due to the critical factors being present in the serum at sufficient concentrations to stimulate NPCs in the FT which possess an innate potential to produce MNs. NPCs from the FT may possess an intrinsic ability to generate cell types (such as MNs) that reside in the spinal cord.

### Hh-Ag 1.3 may be more effective than Shh-N at increasing MN yield from FT-derived neurospheres

In addition to Shh-N, Shh signaling is also activated by the small molecule agonist Hh-Ag 1.3. To generate MNs from ESCs, previous studies have used either 300–500 nM Shh-N or 1–2 µM Hh-Ag 1.3 [54], [67]–[69], [77]. Although Wichterle *et al.* (2002) reported identical results with Shh-N and Hh-Ag 1.3 at these concentrations, most studies have used 1 µM Hh-Ag 1.3 to generate MNs from ESCs [67], [68], [77], [78]. In FT-derived neurospheres, Hh-Ag1.3 was particularly effective in increasing the yield of generated MNs when compared to Shh-N. In the presence of Hh-Ag1.3, nearly 100% of differentiated cells were MNs and this number decreased to 20–40% when Shh-N was used instead. Therefore, a discrepancy exists between the increased efficacy of Hh-Ag 1.3 in FT-derived neurospheres versus ESC-derived neurospheres.

### GFAP^+^ cells derived from FT-derived neurospheres may not be astrocytes

In this study, we used GFAP to identify astrocytes. However, this protein is also a marker for astrocyte-like adult stem cells [61], [79]. In adult mammals, neurogenic astrocytes have been identified *in vivo* in both the SVZ of the lateral ventricle and the subgranular zone of the dentate gyrus in the hippocampus [61], [80]. The characteristics and markers that distinguish neurogenic astrocytes from the vast population of non-neurogenic astrocytes remain unknown [61]. GFAP^+^ cells differentiated from FT-derived NPCs may consist of neurogenic and/or non-neurogenic astrocytes. In our differentiation experiments, cells sometimes double stained for both GFAP and a neuronal marker. For example, FT-derived NPCs that have undergone directed MN differentiation sometimes expressed both MNR2 and GFAP. The concurrent expression of a MN marker with GFAP would be surprising if GFAP were solely a non-neurogenic astrocyte marker. However, because GFAP is also a marker for astrocyte-like adult NPCs, double-stained cells may represent neurogenic astrocytes that are committed to an MN cell fate.

In conclusion, we have demonstrated that FT-derived neurospheres proliferate, can be passaged *in vitro*, and can differentiate into a collection of NPCs, neurons, and glia. The discovery of multipotent cells within the mammalian CNS has had tremendous implications for therapeutic possibilities in many currently incurable CNS diseases, including trauma, Alzheimer's, Parkinson's, ALS, and multiple sclerosis [1], [81]. The establishment of the FT as a source of multipotent cells opens up new possibilities in the field of autologous transplantation therapy for these neurological diseases. It is important to fully explore the potential of these cells both *in vivo* and *in vitro*. The use of a rat model permits a systematic, unlimited study of these cells in a controlled environment. Although cells derived from each animal and even each neurosphere are a heterogeneous population, they all have the ability to divide and to differentiate into both neurons and glia either spontaneously or in a directed manner. Even after these FT-derived cells are frozen for extended periods of time and then subsequently thawed, they still retain the same potential to produce both neurons and glia. This suggests that cells can be collected, frozen, and then later recovered and used for therapeutic purposes. Additionally, because the rat and mouse nervous systems are so closely related, it seems reasonable to expect that the methods that we have developed here will be directly applicable to the mouse. This will allow the use of the large number of already established transgenic lines that have been developed by various stem cell groups. The principles learned from this model may be directly applied to postnatal human FT-derived NPCs for potential therapeutic purposes.
